# Professor Turner

**Published:** 1837-04

**Authors:** 


### OBITUARY.

### PROFESSOR TURNER.

EDWARD TURNER, M.D. F.R.S.L. & E., Professor of Chemistry in University College, London, died on Sunday, 12th February last, at his residence at Hampstead. During some years, Dr. Turner had suffered very much from pain in the right side of the abdomen, for the removal or alleviation of which he had recourse occasionally to local bleeding and blisters. Within the last two years his frame, never robust, became emaciated to such a degree, that, previously to the last fatal illness, all his acquaintances felt astonishment that, though so worn and wasted, he should have never failed to discharge his duties with his characteristic quickness and energy.

In the last days of January, Dr. Turner was seized with influenza, and this disease was the immediate cause of his death. On examination after death, it was found that the lungs were the seat of extensive inflammation (pneumonia), which had its commencement in an attack of the then prevailing epidemic. The cause of the suffering and emaciation of so long continuance was found in the pyloric end of the stomach and in the duodenum. The former was the seat of chronic inflammation and slight ulcerations; the latter was studded with numerous ulcerated points.

Dr. Turner was born in Jamaica, in which island his father possessed considerable property; but at a very early age he was removed to this country. After having received his elementary education at Bath, and studied some time with a surgeon, he was sent to the University of Edinburgh, where he graduated as doctor of medicine. Soon after having taken his degree, he returned to Bath, his English home, with a view to the practice of his profession; but the active, nervous temperament of the young physician could ill brook the tedium of "waiting for practice." In a very short time, therefore, not more than a month we believe, he went to Paris, in company with his contemporary and fellow student, Professor Christison, who had long been, and who remained to the close of life, one of his most intimate and attached friends. It was at Paris, if we mistake not, that Dr. Turner determined to pursue the study of chemistry. In Göttingen he likewise devoted himself most ardently to his favorite science, under the guidance of Professor Stromeyer. In 1824, Dr. Turner began to lecture on chemistry at Edinburgh, and, on the foundation of the University of London, now University College, (1828,) was elected professor of chemistry in that Institution.

As a chemist, Dr. Turner became very early distinguished for the extent and accuracy of his knowledge, and as an acute and original observer. He published several papers in the Transactions of the Royal Societies of London and Edinburgh. The work by which he was most known is the "Elements of Chemistry," which has for some time been, perhaps, the most popular treatise on the subject in this or any other language. Its sale in this country has been very great, (averaging 3,500 in a space of two years,) and its circulation in America has been much more extensive still.

It is impossible to speak truly of Dr. Turner's general character or his moral qualities without running the risk of being accused of exaggeration. He was beloved by all who knew him for the amenity of his manners, the cheerfulness of his disposition, and his enlarged and active benevolence; while he was no less respected for his integrity, his love of justice, and the stainless purity of his life. We hold that almost universally the truest evidence as to the character of a public teacher, whether as an instructor or as a man, may be derived from his pupils; and, applying this test to Dr. Turner, no one could have excelled him.

It would be a culpable omission not to notice the sincere piety and firm christian faith which adorned Dr. Turner, and enabled him to support ill-health, and even to contemplate death, with cheerfulness. This important point of his character was detailed and powerfully dwelt on by his friend and former colleague, the Rev. Thomas Dale, the distinguished Rector of St. Bride's, in an extemporaneous address delivered, at the request of the family, over the grave "when earth was returned to earth;" and subsequently at St. Bride's Church, in an admirable sermon, which we trust will be given to the public in a permanent form.

